# Fortified Blended Food Base: Effect of Co-Fermentation Time on Composition, Phytic Acid Content and Reconstitution Properties

**DOI:** 10.3390/foods8090388

**Published:** 2019-09-03

**Authors:** Ashwini V. Shevade, Yvonne C. O’Callaghan, Nora M. O’Brien, Tom P. O’Connor, Timothy P. Guinee

**Affiliations:** 1 Food Chemistry and Technology, Teagasc Food Research Centre Moorepark, Fermoy, Co. Cork P61 C996, Ireland; 2 School of Food and Nutrition Sciences, University College Cork T12 Y337, Ireland

**Keywords:** fortified blended food base (FBFB), fermented milk, parboiled wheat, phytic acid, consistency

## Abstract

Dehydrated blends of dairy-cereal combine the functional and nutritional properties of two major food groups. Fortified blended food base (FBFB) was prepared by blending fermented milk with parboiled wheat, co-fermenting the blend at 35 °C, shelf-drying and milling. Increasing co-fermentation time from 0 to 72 h resulted in powder with lower lactose, phytic acid and pH, and higher contents of lactic acid and galactose. Simultaneously, the pasting viscosity of the reconstituted base (16.7%, *w*/*w*, total solids) and its yield stress (σ_0_), consistency index (K) and viscosity on shearing decreased significantly. The changes in some characteristics (pH, phytic acid, η_120_) were essentially complete after 24 h co-fermentation while others (lactose, galactose and lactic acid, pasting viscosities, flowability) proceeded more gradually over 72 h. The reduction in phytic acid varied from 40 to 58% depending on the pH of the fermented milk prior to blending with the parboiled cereal. The reduction in phytic acid content of milk (fermented milk)-cereal blends with co-fermentation time is nutritionally desirable as it is conducive to an enhanced bioavailability of elements, such as Ca, Mg, Fe and Zn in milk-cereal blends, and is especially important where such blends serve as a base for fortified-blended foods supplied to food-insecure regions.

## 1. Introduction

Dairy products and cereals constitute major food groups [1] and are frequently combined together to create composite foods. The resultant composites provide a means of innovating products with the additive nutritional, textural and sensory functionalities of both food groups [2,3]. Product examples include yoghurt-cereal bars, and dehydrated milk (fermented milk)-wheat blends including super cereal plus (SCP), kishk and tarhana. Super cereal plus (SCP), a category of fortified blended food supplied by the World Food Programme, is mainly targeted at children 0.5–2 years in food insecure regions. It is typically prepared by dry blending heat-treated wheat/corn/rice (58.3%), dehulled soy beans (20.0%), skim milk powder (8.0%), sugar (9.0%), soy bean oil (3.0%) and vitamin/mineral mix (1.7%) [4]. Kishk and tarhana are dehydrated fermented milk-wheat blends that are widely consumed in the Balkans and Middle East. Kishk is typically formulated by blending yoghurt and parboiled wheat (bulgur) at a weight ratio of 1.5–4:1, incubating (co-fermentation) the blend at 35 °C, shelf-drying to ~7 (%, *w*/*w*) moisture (traditionally the product was sun dried) and milling [5,6]. Tarhana is similar to kishk except that parboiled wheat is substituted with wheat flour, baker’s yeast (*Saccharomyces cerevisiae*), diced vegetables and spices are optionally added [7]. Dehydrated milk-cereal blends are typically reconstituted and consumed as a nutritious porridge or soup [4,8]. 

In the traditional manufacture of kishk and tarhana, incubation occurred over a period of 4–7 days [5,9]. This probably reflects the widespread production at domestic level, whereby the cereal and milk (fermented by back-slopping) were mixed, held (incubated) at high ambient temperatures (30–45 °C) and frequently kneaded until the blend acquired a dough-like consistency, which enabled it to be formed into balls, nuggets or layers that could be easily sun-dried prior to size reducing (grinding) into a powder. Undoubtedly, such a process was conducive to variation in composition and pH of the dried blend. The use of fermented milk rather than milk or dehydrated skim milk powder (as in Super Cereal Plus) improves the potential nutritional value of dairy-cereal blends by increasing the content of bioactive compounds and B vitamins [10,11] and reducing the level of lactose [12]. Moreover, co-fermentation of the milk and cereal has the potential to reduce the anti-nutrient effects of phytic acid (myoinositol 1,2,3,4,5,6-hexakis dihydrogen phosphate), and thereby, to increase mineral bioavailability [2,9,10]. Phytic acid is a highly negatively charged ion that can bind cationic salts (elements) and proteins to form insoluble complexes, thereby rendering them less bioavailable in the gastrointestinal tract [13,14,15]. The production of lactic acid reduces the pH to values (5.0–5.5) more favourable to the activity of endogenous cereal phytases, which hydrolyse the phosphate from phytic acid and, thereby, reduce their nutrient binding effects [16,17]. Hence, the co-fermentation of fermented milk-wheat blends offers an alternative approach for the development of a fortified blended food base (FBFB) with enhanced biofunctionality.

Reducing the co-fermentation time of dairy-cereal blend would be more amenable to commercial manufacture of FBFB, and reduce the risk of product contamination (e.g., with yeasts, moulds) and quality deterioration of the resultant FBFB on reconstitution [6]. Erbaş et al. [18] reported that increasing the co-fermentation time of a wet tarhana blend from 0 to 72 h at 25 °C led to reductions in the populations of lactic acid bacteria, aerobic mesphophiles, yeasts and moulds; simultaneously, the concentrations of lactic, acetic, propionic and pyruvic increased, while pH decreased. Ekinci [19] showed that incremental extension of co-fermentation time from 0 to 96 h at 35 °C coincided with significant increases in the levels of riboflavin, pantothenic acid, folic acid and ascorbic acid in tarhana but had no effect on contents of thiamine or pyridoxine. Increasing co-fermentation time has also been found to reduce the phytic acid content of kishk and tarhana powders to a degree dependent on formulation and fermentation time [9,20]. We are unaware of studies on the impact of co-fermentation time on lactose metabolism in kishk or on its consistency when reconstituted. While the nutritional value of semi-liquid foods such as porridge and soup are primarily determined by composition, it is also likely to be influenced by texture and viscosity, which affect satiety, rate of gastric emptying and sensory appeal [21,22,23].

The objective of the current study was to evaluate the effect of co-fermentation time on the composition, phytic acid content and the reconstitution properties of an FBFB prepared from fermented milk and parboiled wheat. 

## 2. Materials and Methods

### 2.1. Ingredients

Buttermilk powder (protein 33%, fat 7%, lactose 46%, lactic acid 0.23%) was procured from Glanbia Ingredients Plc. (Ballyragget, Co. Kilkenny, Ireland). Low-heat skim milk powder (protein 38.43%, fat 0.89%, lactose 46.2%, lactic acid 0.04%) was manufactured using a pilot-scale NIRO Tall-Form Dryer. Cream (fat 37%) was purchased from a local retail store. Freeze-dried direct-vat-set starter cultures CH1 YoFlex^®^ 207 (*Streptococcus thermophilus*) and YC380 (*Lactobacillus delbrueckii* subsp. *bulgaricus*) were purchased from Chr. Hansen Ireland Ltd. (Rohan Industrial Estate, Little Island, Co. Cork, Ireland). 

### 2.2. Preparation of Fermented Milk (FM) and Parboiled Wheat (PW)

FM and PW were prepared as described previously by Shevade et al. [24]. Briefly, reconstituted milk was prepared by dispersing skim milk powder, buttermilk powder and cream, at levels of 7.0, 8.9 and 4.5% (*w*/*w*) in water. The reconstituted milk was heat treated (95 °C for 2.5 min), homogenised at first and second-stage pressures of 15 and 5 MPa, and cooled to 42 °C (UHT/HTSTLab-25 EHVH, MicroThermics^®^, Raleigh, NC, USA). The pasteurised milk (20 L) was then inoculated with starter cultures CH1 YoFlex^®^ and YC380 at respective levels of 19.6 and 20.2 culture units per 100 L, incubated at 42 °C (Heratherm™ Advance Protocol Microbiological Incubators, Thermo Scientific™, Waltham, MA, USA) until pH decreased to 4.6 (unless otherwise stated), stirred for 30 min at 120 rpm (Model RW 16, IKA Werke GmbH, Staufen im Breisgau, Germany) while cooling in an ice-bath to 15 °C, and stored overnight at 4 °C. The pH of FM did not drop further during cooling and overnight storage at 4 °C. In a separate set of experimental designed to investigate the effect of the pH of the FM on the phytic acid content of the FBFB powders, the FM was cooled to 15 °C, when the pH value had reached 5.6, 5.2, 5.0, 4.6 or 4.2 and immediately blended with PW in the manufacture of the FBFB, as described below. 

PW was prepared by adding dehusked wheat kernel to deionized water (at 1.5 kg/kg kernel), heating the mixture at 90 °C for 60–70 min, drying at 52 °C for 24 h and milling to 1 mm (Ultracentrifugal Mill ZM 200, Retsch Technology GmbH, Haan, Germany).

### 2.3. Preparation of Fortified Blended Food Base (FBFB)

FBFBs were prepared on three separate occasions as described previously [24]. Essentially, FM and PW were blended at weight ratio of 3.4:1.0 (Kenwood blender, Model KMM710 fitted K-beater; Kenwood Ltd., Fareham, Hampshire, UK). The resultant blend was divided into four 2 kg sub-batches, incubated for 0, 24, 48 or 72 h at 35 °C (Heratherm Incubator, ThermoFisher Scientific, Waltham, MA, USA), rolled into thin layers (30 cm × 30 cm × ~0.5–1.0 cm) and dried for 48 h at 46 °C (Excalibur^®^ Dehydrator, Sacramento,CA, USA); the resultant ‘cake’ was size reduced (Hallde RG-350 machine, AB Hallde Maskiner, Kista, Sweden) and milled to 1 mm, vacuum packed in polythene liners (PE60/Met polyester) and stored in a temperature-controlled powder room at 15 °C until analysed.

FBFB produced using fermentation times of 0, 24, 48 or 72 h at 35 °C were denoted FBFB0, FBFB24, FBFB48 and FBFB72, respectively.

### 2.4. Composition of FBFB Powders

Powders were analysed in triplicate for protein by Leco nitrogen analyser (Model FP268, Leco Corporation, Michigan, MI, USA), fat by CEM Smart Trac (Cem Corporation, Matthews, NC, USA), NaCl by potentiometric determination of chloride, moisture by drying to constant weight at 102 °C, and pH was measured on a 5% aqueous dispersion of the FBFB, prepared by stirring for 15 min at 21 °C (inoLab 7310 pH meter, WTW GmbH, Weilheim, Germany) as described previously [24]. The concentrations of lactose, lactic acid, starch, glucose, maltose and phytic acid were measured using the Megazyme kit K-LACGAR, K-DLATE, K-TSHK 09/15 kit, K-MASUG 08/18 and K-PHYT 05/17, respectively (Megazyme International Ireland, Bray Business Park, Bray, Co. Wicklow, Ireland).

### 2.5. Microbiology of the FBFB Wet Blend and Powders

The fermented milk, wet FBFB and dried FBFB powders were assayed in duplicate for *Sc. thermophilus* and *Lb. delbrueckii* subsp. *bulgaricus* [25]. Each material was diluted 1:10 in sterile maximum recovery diluent (MRD), homogenised using stomacher (Stomacher, Lab-Blender 400, IUL, S.A., Barcelona, Spain) for 5 min, and serially diluted 1:10 in MRD up to eight times. *Sc. thermophilus* were enumerated on LM-17 agar plates incubated aerobically at 42 °C for 72 h, and *Lb. delbrueckii* subsp. *bulgaricus* on MRS agar plates incubated anaerobically at 37 °C for 72 h. Counts were expressed as log_10_ cfu/g.

### 2.6. Analysis of Reconstituted FBFB Powder (R-FBFB)

#### 2.6.1. Gelatinization temperature

Gelatinization temperature was determined using differential scanning calorimetry (DSC 2000, TA instruments, New Castle, DE, USA), as described previously [24]. FBFB samples (1.0–1.4 g) were reconstituted in distilled water at 20 °C to a fixed (30:1) water-to-starch ratio, stirred for 15 min at 500 rpm. A sub-sample (20–30 mg) was sealed hermetically in an aluminium differential scanning caloriemetry Tzero pan, equilibrated at 20 °C, and heated to 95 °C at 5 °C/min; an empty pan was used as a reference.

#### 2.6.2. Water Holding Capacity (WHC)

FBFB powder was reconstituted in deionized water (16.7%, *w*/*w*) and placed in doubled-jacketed, capped glass vessel (Therm 500 mL, Product No. 61418250, Metrohm Ireland Ltd., Carlow, Ireland) fitted with an overhead stirrer (Model RW16, IKA Werke GmbH, Staufen im Breisgau, Germany) and connected to a thermostatically-controlled water bath (Model Julabo EH-5,Julabo GmbH, Seelbach, Germany). The sample was heated to 95 °C over 10 min, held at 95 °C for 25 min and cooled to 25 °C in ice water bath with continuous stirring over 10 min [24]. The cooled sample was centrifuged at 12,500× *g* for 1 h at 20 °C (Sorvall Lynx 6000 Superspeed centrifuge, ThermoElectron LED GmbH, Langenselbold, Germany). WHC was defined as weight of pellet per 100 g of R-FBFB.

#### 2.6.3. Soluble Starch (SSC)

R-FBFB samples (16.7%, *w*/*w*) were cooked and cooled, as described for WHC measurement. The cooled sample was centrifuged at 12,500× *g* for 1 h at 20 °C. A sample of the supernatant (10 mL) was analysed for SSC using the Megazyme enzymatic kit K-TSHK 09/15 (Megazyme International Ireland, Bray Business Park, Bray, Co. Wicklow, Ireland). SSC was defined as the starch content of the supernatant expressed as a percentage of the starch in the R-FBFB prior to centrifugation [26].

#### 2.6.4. Pasting Behaviour

A sample (15 g) of the R-FBFB (16.7%, *w*/*w*) was placed in starch pasting cell fitted to a controlled stress rheometer (Anton Paar Physica MCR 501 Rheometer, Anton Paar GmbH, Graz, Austria), tempered at 25 °C for 1 min, heated to 95 °C over 10 min, held at 95 °C for 25 min, and cooled to 25 °C for 10 min while constantly shearing at 160 s^−1^ [24]. The following parameters were obtained from the resultant viscosity/time curve: viscosity after heating to 95 °C (V_95_), peak viscosity on holding at 95 °C (V_p_), viscosity after holding at 95 °C (V_h_) and cooling to 25 °C (V_c_), breakdown viscosity (BRV) and setback viscosity (SBV), corresponding to the viscosity drop during holding at 95 °C and viscosity increase on cooling, respectively.

#### 2.6.5. Rheology

The R-FBFB (16.7%, *w*/*w*) was cooked (as described for WHC) and cooled to 60 °C to simulate the temperature at which the cooked R-FBFB powder is typically consumed. A subsample (11 g) was placed in the measuring cell of a controlled stress-rheometer (Carri-Med, type CSL2500, TA instruments, New Castle, DE, USA), equilibrated at 60 °C for 5 min, and subjected to shear rate ($\dot{\gamma }$) sweep from 18 to 120 s^−1^ over a period of ~20 min. Shear stress (σ; Pa) and viscosity (η; Pa·s) were measured as function of $\dot{\gamma }$ [24]. The resultant σ/$\dot{\gamma }$ data were fitted to the Herschel-Bulkley model using TA data analysis software (TA Rheology Advance Data Analysis, Version V5.7.0, New Castle, DE, USA):

(1) $$\sigma \;=\;\sigma_{0}\;+\;K\;{\dot{\gamma }}^{n}$$

where σ_0,_ K and n represent yield stress (Pa), consistency coefficient (Pa.s) and flow behaviour index (*n*), respectively. In addition to σ_0_, K and n, the final viscosity after shearing (η_120_) was also recorded. 

#### 2.6.6. Flowability

Samples of the R-FBFB were cooked (as described for WHC), cooled to 46 °C, and evaluated for flowability using a Bostwick Consistometer (CR Instruments Limited, Bournemouth, UK), as described previously [26]. Flow was expressed in mm/30 s [4].

### 2.7. Statistics

A randomized complete block design incorporating the four treatments (FBFB0, FBFB24, FBFB48 and FBFB72) and three blocks (replicate trials) was used for the analysis of response variables. The effects of treatment were determined using analysis of variance (ANOVA), carried out using the general linear model procedure of SAS 9.3 [27]. Tukey’s multiple-comparison test was used for paired comparison of treatment means and the level of significance was determined at *p* < 0.05.

Simple linear regression was carried out using Microsoft Excel 2010 to determine Pearson correlation between the different response variables, where significance was determined at *p* < 0.05, according to Student’s *t*-test.

## 3. Results

### 3.1. Changes in Starter Bacteria During Co-Feremntation and Drying 

**Figure 1:**
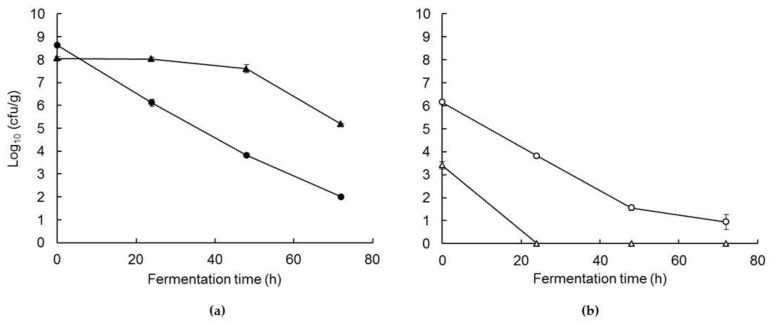
Effect of fermenation time on the counts of *Sc. thermophilus* (●,○) and *Lb. delbruckeii* subsp. *bulgaricus* (▲,△) in the fermented milk wheat blend before (**a**) and after (**b**) drying.

The mean count of *Sc.thermophilus* and *Lb. delbrueckii* subsp. *bulgaricus* increased from 10^6.1^ and 10^7.1^ cfu/g, respectively, in the milk immediately after inoculation, to 10^9.3^ and 10^8.6^ cfu·g/g in the fermented milk. Blending of the FM with the parboiled wheat (PW) at a weight ratio of 3.4:1 reduced the counts of *Sc.thermophilus* and *Lb. delbrueckii* subsp. *bulgaricus* to ~22.5 and 26% of original values, i.e., to 10^8.6^ and 10^8.1^ cfu/g, respectively. The counts of both starter cultures in the wet FBFB blend decreased progressively with fermentation time to 10^2.0^ and 10^5.2^ cfu·g^−1^, respectively, after 72 h (Figure 1a). This trend most likely reflects a reduction in starter culture viability due an increase osmotic stress associated with the increase in the concentrations of glucose and galactose, and the continued reduction in pH [28]. The populations of both starter cultures decreased to <10% of the corresponding counts in the wet blends on drying at 46 °C for 48 h (Figure 1b). The reduction in starter culture viability during drying of the blend is consistent with the results of previous studies on spray drying yoghurt [29,30].

### 3.2. Properties of the FBFB Powders

#### Composition and pH

The mean compositions of FBFBs are shown in Table 1. The compositional parameters were within the ranges previously reported for dehydrated fermented milk-cereal blends, i.e., 35–45.2% (*w*/*w*) starch, 14–25% (*w*/*w*) protein, 3.6–10.7% (*w*/*w*) fat, 2–10% (*w*/*w*) lactose, 1.8–4.6% (*w*/*w*) lactic acid and pH 3.9–5.2 [6,8]. 

Increasing co-fermentation time from 0 to 72 h had no effect on the contents of protein, fat, starch or salt (*p* > 0.05), but significantly affected the quantities of sugars (lactose, galactose), lactic acid and pH. The lactose content and pH decreased progressively with fermentation time, from 6.82% (*w*/*w*) and 4.14, respectively, at 0 h to 4.54% (*w*/*w*) and 3.85 at 72 h; simultaneously, the levels of galactose and lactic acid increased. The presence of unfermented lactose (4.54–5.71%, *w*/*w*) after 24–72 h fermentation time suggests inhibition of the starter cultures because of the low pH of the wet base prior to drying [28], i.e., pH 4.4, 3.9, 3.8 and 3.7 after 0, 24, 48 and 72 h fermentation (data not shown). The increase in galactose content with fermentation time and the very low level of glucose (<0.1%) at all fermentation times indicates that both starter cultures were galactose-negative, and fermented the glucose moiety of lactose only [31]. The reduction in pH from 4.14 in FBFB0 to 3.85 in FBFB72 was similar in magnitude to that reported in previous studies [32]. 

**Figure 2:**
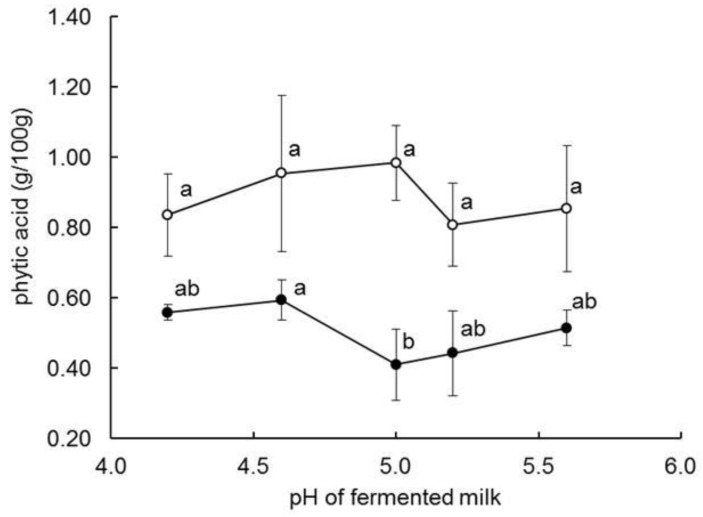
Effect of pH of fermented milk on the phytic acid content of fortified belnded food base prepared from fermented milk and parboiled wheat; prior to drying, the wet FBFB was incubated at 35 °C for 0 (○) or 24 (●) h. pH abscissae along each curve not sharing a common lower-case superscipt differ significantly (*p* < 0.05) in phytic acid content.

The phytic acid content (%, *w*/*w*) of the powders (~ 0.53–0.87, Table 1) was within the range of values previously reported for commercial kishk (0.43–0.68, [33]) and experimental fermented milk-wheat bulgur blends (0.37–0.85, [9,33]). Phytic acid content (*w*/*w*) decreased by ~40% on increasing fermentation time from 0 h (FBFB0) to 24 h (FBFB24) (*p* < 0.05), and remained essentially constant on extending the fermentation time further to 48 or 72 h (*p* > 0.05). In a further experiment, where the pH of the FM was varied from 4.2–5.6 prior to blending with the PW, the reduction in phytic acid content after 24 h fermentation decreased from 58% at pH 5.0 to 33% at pH 4.2 (Figure 2). The lower phytic acid content of FBFB made from FM at pH 5.0, as opposed to lower, or higher, pH values, is consistent with the occurrence of the pH optimum of endogenous wheat phytase at 5.0–5.5 [34]. Compared to the current results, Toufeili et al. [9] reported a more gradual reduction in the phytic acid content of a dehydrated yoghurt-bulgur wheat blend with fermentation time, i.e., to 25 and 40% of original values after 24 and 96 h, respectively. Bilgiçli and İbanoğlu [20] reported a considerably higher reduction in the phytic acid content of tarhana, i.e., ~91% after 72 fermentation. The inter-study difference in the extent of phytic acid reduction with fermentation time may reflect differences in phytic content of the wheat cultivar used [34,35], differences in parboiling and milling conditions [36], pH of the milk/fermented milk, and/or the rate of drying.

### 3.3. Properties of Reconstituted Fortified Blended Food Base (R-FBFB)

#### 3.3.1. Gelatinization, Water holding Capacity and Starch Solubilisation on Cooking and Cooling 

None of the R-FBFBs underwent gelatinization, as indicated by the absence of an endothermic phase transition peak on heating from 20 to 95 °C (data not shown). The result concurs with previous studies [37,38], which showed that parboiling (e.g., ~95 °C for ~70 min in current study) of cereal promoted pre-gelatinization of starch granules, with a reduction in the degree of starch crystallinity and loss of the DSC heat endotherm during subsequent heating.

The WHC of the reconstituted R-FBFB increased during pasting (heating and holding), and remained relatively constant during cooling (Table 2). These changes reflect water uptake by, and concurrent swelling of, the starch granules [39]. WHC was unaffected by fermentation time. While the WHC of the R-FBFB is most likely dominated by the starch component [24,40], it is probably also affected by the presence of other compositional factors, e.g., types and concentrations of salts [41,42] and lactic acid [43]. Soluble starch content (SSC) increased progressively during pasting and cooling (Table 2), suggesting a progressive dissociation of starch molecules from the granule to the surrounding aqueous phase and a simultaneous loss of granule integrity. The mean SSC of FBFB72 was slightly, but significantly, higher than that of FBFB0 at the end of pasting (35 min) and cooling (45 min). The data suggest that prolonging the fermentation time from 0 to 72 h rendered the starch granule more sensitive to fragmentation during pasting and cooling of the R-FBFB [39]. Linear regression analysis showed that SSC at 35 and 45 min correlated positively with lactic acid content and negatively with pH, which increased and decreased respectively with fermentation time (Table 3). 

The positive correlation between SSC and lactic acid content concurs with the findings of Majzoobi and Beparva [43] which showed that acidification of wheat-starch suspensions in the pH range (4.24–3.34) increased the starch solubility index after heating to 90 °C.

#### 3.3.2. Pasting Behaviour

**Figure 3:**
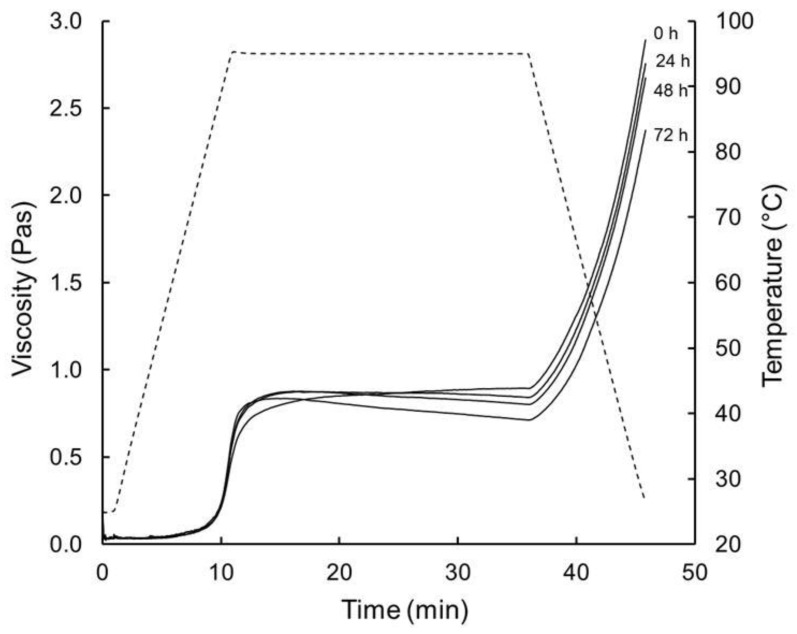
Changes in viscosity (solid lines) and temperature (broken line) during pasting (heating and holding at 95 °C) and cooling of reconstituted fortified blended food base (R-FBFB) prepared using different co-fermentation times: 0 h (FBFB0), 24 h (FBFB24), 48 h (FBFB48) or 72 h (FBFB72).

The viscosity of all R-FBFB increased on reaching to ~90–95 °C (Figure 3). For R-FBFB24, R-FBFB48 and R-FBFB72, it increased further to a peak value (V_p_) on holding at 95 °C for 5–6 min and then decreased until the end of the holding period. In contrast, the viscosity of R-FBFB0 increased continuously on holding at 95 °C and did not display a V_p_ value. All FBFBs exhibited an increase in viscosity on cooling to 25 °C. The viscosity changes of R-FBFB24, R-FBFB48 and R-FBFB72 on pasting and cooling are typical of those previously reported for aqueous dispersions of starch [39,44,45] and fermented milk-wheat blends [7,26]. 

The viscosity increase during heating and the initial stages of holding reflects water uptake by, and concomitant swelling of, the starch granules, while the viscosity reduction on prolonged holding coincides with granule fracture/collapse, associated with dissociation of hydrated starch molecules from the granule. Starch dissociation was confirmed by the increase in SSC in all FBFB during cooking and cooling (Table 2). The viscosity increase on cooling (SBV) has been attributed to re-association of solubilised amylose molecules [46,47].

Similar to the trend for SSC, increasing co-fermentation time from 0 to 48 h had little, or no, effect on viscosity at the different stages of pasting and cooling, as evidenced by the similar values of V_95_, V_p_, V_c_, BRV and SBV values of R-FBFB0, R-FBFB24 and R-FBFB48 (Table 2). In contrast, extending co-fermentation time from 0 to 72 h resulted in significantly higher values of V_95_ and BRV, and lower values of V_h_, V_c_ and SBV (*p* < 0.05). The lower V_h_ and V_c_ values of R-FBFB72 are consistent with its higher SSC at 35 and 45 min [39]. V_95_ correlated positively with concentrations of lactic acid and galactose and negatively with pH and lactose content (Table 3); an opposite effect was found for V_h_ and V_c_. These trends suggest that lactic acid renders the starch granule more amenable to swelling during the heating but more susceptible to rupture on prolonged holding at 95 °C [44]. Analogous to the current trend for V_h_ and V_c_, Hirashima et al. [48] reported that the shear viscosity of heated (95 °C) and cooled (25 °C) corn starch suspensions (3%, *w*/*w*) decreased steeply with incremental reduction of pH from 4.0 to 3.0 by the of addition organic acids prior to heating, and attributed this to the hydrolysis of amylose and amylopectin. Likewise, Ohishi et al. [44] found that acidification of rice starch suspensions (4%, *w*/*w*) with acetic acid (0.0–0.2 M) led to incremental reduction in pH (6.8–3.0) and viscosity after holding (95 °C) and cooling (50 °C).

#### 3.3.3. Rheology and Flowability

**Figure 4:**
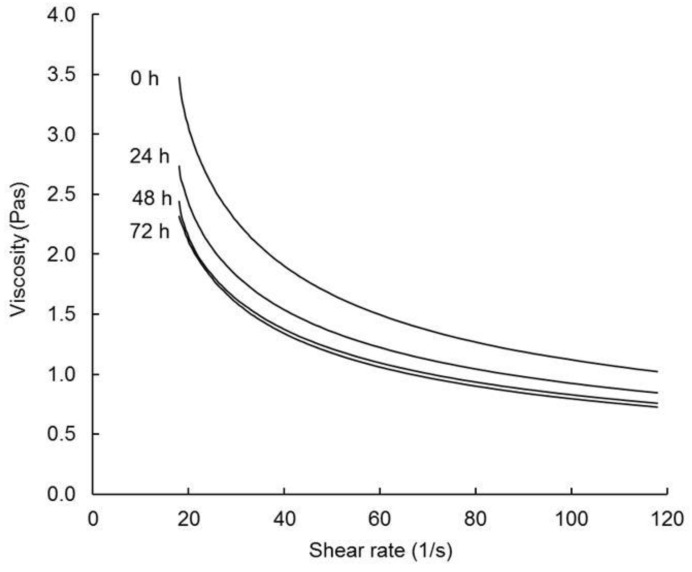
Flow curves at 60 °C of reconstituted fortified blended food base (R-FBFB) manufactured using different co-fermentation times: 0 h (FBFB0), 24 h (FBFB24), 48 h (FBFB48) or 72 h (FBFB72).

Following cooking, the R-FBFBs were cooled to 60 °C and subjected to a shear rate sweep from 18 to 120 s^−1^. All products displayed a yield stress (σ_0_) and shear thinned (Figure 4) with increasing shear rate ($\dot{\gamma }$), indicating disruption of an internal network, most likely comprised of a complex of starch granules and protein which dominate the matrix [26]. 

A similar trend was reported in previous studies for reconstituted fermented milk-wheat blends [26,49]. Viscosity over the complete shear rate range decreased in the following order: R-FBFBo > R-FBFB24 > R-FBFB48 ≈ R-FBFB72 (Figure 4). R-FBFB0 had significantly higher yield stress (σ_0_) and final viscosity (η_120_) than all other FBFB, and a higher consistency index (K) than R-FBFB72 (Table 2). Similar to the trends for the pasting parameters, V_h_ and V_c_, the values of σ_0_, K and η_120_ correlated negatively with levels of lactic acid in FBFB powders, and with SSC of the R-FBFB after cooking (SCC35 min) and cooling (SCC45 min) (Table 3). 

The flowability of all R-FBFBs exceeded the minimum (≥100 mm/30 s; Table 2) as specified by the WFP for porridge prepared from reconstituted Super Cereal plus [4]. The flowability of R-FBFB48 and R-FBFB72 were significantly higher than that of R-FBFB24 or R-FBFB0. The higher flowability of R-FBFB48 and R-FBFB72 is consistent with their lower viscosity after shearing (η_120_). The variation in the consistency of the R-FBFB with co-fermentation time may provide a basis for altering sensory properties [50], satiety value [51] and glycaemic index [21,52]. 

## 4. Conclusions

Fortified blended food base (FBFB) was formulated by blending fermented milk and parboiled wheat, co-fermenting the wet blend at 35 °C, drying at 46 °C and milling the resultant cake to a powder of 1 mm particle size. Increasing co-fermentation time resulted in powders with lower contents of lactose and phytic acid, lower pH and higher levels of lactic acid and galactose. Simultaneously, the reconstituted base became less viscous and thinner after cooking (V_h_) and cooling (V_c_). Co-fermentation time affected evaluated characteristics differently, whereby changes in some characteristics (e.g., pH, phytic acid, η_120_) were essentially complete after 24 h co-fermentation, while others (e.g., concentrations of lactose, galactose and lactic acid, pasting viscosities, flowability) proceeded more gradually over 72 h of co-fermentation. The reduction in phytic acid content (as a % of the level prior to co-fermentation) during the first 24 h of co-fermentation varied with the pH of the fermented milk prior to blending with the parboiled wheat, i.e., from ~40% at pH 4.4–4.6 to 58% at pH 5.0. The reduction in phytic content with co-fermentation enhances the potential bioavailability of elements, such as Ca, Mg, Fe and Zn, in milk-cereal blends, and is especially important where such blends serve as the base for fortified-blended foods supplied to food-insecure regions. Our results showed that co-fermentation time during the manufacture of FBFB could be reduced from 72–96 h (as practiced traditionally in the manufacture of fermented milk-cereal blends) to 24 h, without affecting phytic acid content; moreover, it enhanced the viscosity and thickness of the reconstituted base. 

## Figures and Tables

**Table 1:** Composition of fortified blended food base (FBFB) prepared using different co-fermentation time.

Item		Fermentation Time			
	Code:	0 FBFB0	24 FBFB24	48 FBFB48	72 FBFB72
Powder composition					
Dry matter (%, *w*/*w*)		93.83 ^a^	93.44 ^a^	93.65 ^a^	93.21 ^a^
Moisture (%, *w*/*w*)		6.17 ^a^	6.56 ^a^	6.35 ^a^	6.79 ^a^
Protein (%, *w*/*w*)		19.05 ^a^	18.88 ^a^	19.05 ^a^	19.19 ^a^
Fat (%, *w*/*w*)		6.09 ^a^	6.07 ^a^	6.23 ^a^	6.01 ^a^
Salt (%, *w*/*w*)		0.56 ^a^	0.59 ^a^	0.64 ^a^	0.62 ^a^
Starch (%, *w*/*w*)		37.33 ^a^	36.36 ^a^	38.33 ^a^	37.41 ^a^
Lactose (%, *w*/*w*)		6.82 ^a^	5.43 ^b^	5.71 ^bc^	4.54 ^c^
Galactose (%, *w*/*w*)		3.60 ^b^	4.18 ^ab^	4.21 ^ab^	4.80 ^a^
Lactic acid (%, *w*/*w*)		3.75 ^c^	4.47 ^b^	4.87 ^ab^	5.01 ^a^
Glucose (%, *w*/*w*)		0.09 ^a^	0.06 ^b^	0.05 ^b^	0.05 ^b^
Maltose (%, *w*/*w*)		0.00 ^a^	0.00 ^a^	0.00 ^a^	0.00 ^a^
Phytic acid (%, *w*/*w*)		0.87 ^a^	0.53 ^b^	0.56 ^b^	0.53 ^b^
pH		4.14 ^a^	3.94 ^b^	3.88 ^b^	3.85 ^b^

^a–c^ Values within a row relating to FBFB prepared using different fermentation times and not sharing a common lower-case superscripted letter differ significantly (*p* < 0.05). Presented data are mean values for three replicate trials.

**Table 2:** Reconstitution characteristics of fortified blended food base (FBFB) prepared using different co-fermentation times. ^1,2.^

Item		Co-Fermentation Time (h)			
	Code:	0	24	48	72
		R-FBFB0	R-FBFB24	R-FBFB48	R-FBFB72
Water holding capacity (WHC; g pellet/100 g)					
0 min		43.9 ^a^	43.7 ^a^	43.3 ^a^	42.9 ^a^
10 min		60.9 ^a^	60.5 ^a^	60.5 ^a^	60.3 ^a^
35 min		75.9 ^a^	75.4 ^a^	75.2 ^a^	74.8 ^a^
45 min		75.4 ^a^	75.6 ^a^	75.7 ^a^	75.3 ^a^
Soluble starch (SSC, % total starch)					
0 min		7.6 ^a^	7.4^a^	7.7 ^a^	7.8 ^a^
10 min		28.9 ^a^	29.3^a^	29.3 ^a^	32.0 ^a^
35 min		38.2 ^b^	38.2^b^	41.0 ^ab^	41.1 ^a^
45 min		40.4 ^b^	42.3^ab^	43.5 ^ab^	44.9 ^a^
Pasting/cooling viscosity ^2^					
V_95_ (Pas)		0.53 ^b^	0.59 ^ab^	0.62 ^ab^	0.66 ^a^
V_p_ (Pas)		-	0.86 ^a^	0.88 ^a^	0.90 ^a^
V_h_ (Pas)		0.90 ^a^	0.84 ^ab^	0.81 ^ab^	0.71 ^b^
V_c_ (Pas)		2.91 ^a^	2.74 ^ab^	2.70 ^ab^	2.37 ^b^
BRV (Pas)		−0.10 ^b^	0.02 ^ab^	0.07 ^a^	0.18 ^a^
SBV (Pas)		2.01 ^a^	1.91 ^ab^	1.89 ^ab^	1.66 ^b^
Rheology ^2^					
σ_o_ (Pa)		29.4 ^a^	22.7 ^b^	22.2 ^b^	20.5 ^b^
K (Pa·s^n^)		14.3 ^a^	11.7 ^ab^	11.8 ^ab^	10.4 ^b^
*n* (-)		0.70 ^a^	0.66 ^a^	0.65 ^a^	0.63 ^a^
η at 120 s^−1^ (Pas)		1.1 ^a^	0.8 ^b^	0.8 ^b^	0.7 ^b^
Flowability (mm/30 s)		100.2 ^b^	100.2 ^b^	110.2 ^a^	110.7 ^a^

^a–b^ Values within a row relating to R-FBFB prepared using different times and not sharing a common lower-case superscripted letter differ significantly (*p* < 0.05). ^1^ Presented data are mean values for three replicate trials. ^2^ Abbreviations: R-FBFB, reconstituted FBFB; V_95_, V_h_ and V_c_ denote viscosity on heating to 95 °C, after holding at 95 °C for 25 min, and after cooling to 30 °C, respectively; V_p_, BRV and SBV correspond to peak viscosity during heating and holding, viscosity decrease on holding at 95 °C (V_p_–V_h_) and viscosity increasing during cooling (V_c_–V_h_), respectively; σ_o_, K, *n* and η_120_ refer to yield stress, consistency index, flow behaviour index and viscosity at 120 s^−1^, respectively, on shearing the cooled R-FBFB from 20 to 120 s^−1^ at 60 °C.

**Table 3:** Correlation coefficients between composition of fortified blended food base (FBFB) and the characteristics of the reconstituted base (R-FBFB) ^1,2^.

Characteristic of R-FBFB	FBFB Composition			
	Lactose (%, *w*/*w*)	Lactic Acid (%, *w*/*w*)	Galactose (%, *w*/*w*)	pH
Water holding capacity (WHC; g pellet/100 g) at				
0 min	0.26	−0.19	−0.4	0.80 ***
10 min	0.13	−0.04	−0.2	0.10
35 min	0.33	−0.28	−0.4	0.10
45min	−0.02	0.03	−0.1	0.30
Soluble starch content (% total starch) at				
0 min	0.00	0.20	0.00	−0.20
10 min	−0.20	0.20	0.20	−0.30
35 min	−0.50	0.70 **	0.50	−0.70 **
45 min	−0.76 **	0.78 **	0.8 ***	−0.80 ***
Pasting/cooling viscosity ^2^				
V_95_ (Pas)	−0.75 **	0.75 **	0.70 **	−0.70 **
V_p_ (Pas)	0.23	0.33	0.51	0.01
V_h_ (Pas)	0.75 **	−0.66 *	−0.60 *	0.70 **
V_c_ (Pas)	0.74 **	−0.59 *	−0.60 *	0.60 *
BRV (Pas)	−0.21	0.26	0.10	−0.40
SBV (Pas)	0.72 **	−0.55 *	−0.60 *	−0.60 *
Rheology ^2^				
σ_o_ (Pa)	0.86 ***	−0.78 **	0.8 ***	0.80 ***
K (Pa·s^n^)	0.65 *	−0.54	0.8 ***	0.80 ***
*n* (-)	0.64 *	−0.6 **	0.6 **	0.60 *
η at 120 s^−1^ (Pas)	0.81 ***	−0.84 ***	0.9 ***	0.90 ***
Flowability (mm/30 s)	−0.56 *	0.63 *	0.56 *	−0.62 *

^1^ Correlation coefficients obtained using simple linear regression analysis of the entire data set, relating to four treatments of FBFB prepared on three different occasions. Significance levels: ***, *p* < 0.001; **, *p* < 0.01; *, *p* < 0.05. Negative correlations indicated by a negative sign (−). ^2^ Abbreviations: R-FBFB, reconstituted FBFB;V_95_, V_h_ and V_c_ denote viscosity on heating to 95 °C, after holding at 95 °C for 25 min and after cooling to 30 °C, respectively; V_p_, BRV and SBV correspond to peak viscosity during heating and holding, viscosity decrease on holding at 95 °C (V_p_–V_h_) and viscosity increasing during cooling (V_c_–V_h_), respectively; σ_o_ K, *n* and η_120_ refer to yield stress, consistency index, flow behaviour index and viscosity at 120 s^−1^ respectively, on shearing the cooled R-FBFB from 20 to 120 s^−1^ at 60 °C.
